# Cortical microvascular raspberries and ageing: an independent but not exclusive relationship

**DOI:** 10.1186/s40478-023-01700-z

**Published:** 2023-12-12

**Authors:** Henric Ek Olofsson, Thea Österling Delshammar, Elisabet Englund

**Affiliations:** Division of Pathology, Department of Clinical Sciences Lund, Lund University, Sölvegatan 25 B, 22185 Lund, Sweden

**Keywords:** Cerebrovascular disease, Small vessel disease, Aging, Hypertension, Atherosclerosis, Cardiac hypertrophy, Brain ischemia, Cerebral angiogenesis, Cerebral neovascularization

## Abstract

**Introduction:**

Raspberries are cerebral microvascular formations of unknown origin, defined as three or more transversally sectioned vascular lumina surrounded by a common perivascular space. We have previously demonstrated an increased raspberry density in the cortex of patients with vascular dementia and cerebral atherosclerosis, while studies by other authors on overlapping and synonymously defined vascular entities mainly associate them with advancing age. The aim of the present study was to examine the relationship between raspberries and age in a large study sample while including multiple potential confounding factors in the analysis.

**Materials and methods:**

Our study sample consisted of 263 individuals aged 20–97 years who had undergone a clinical autopsy including a neuropathological examination. The cortical raspberry density had either been quantified as part of a previous study or was examined de novo in a uniform manner on haematoxylin- and eosin-stained tissue sections from the frontal lobe. The medical records and autopsy reports were assessed regarding neurodegeneration, cerebral infarcts, cerebral atherosclerosis and small vessel disease, cardiac hypertrophy, nephrosclerosis, hypertension, and diabetes mellitus. With the patients grouped according to 10-year age interval, non-parametric tests (the Kruskal–Wallis test, followed by pairwise testing with Bonferroni-corrected *P* values) and multiple linear regression models (not corrected for multiple tests) were performed.

**Results:**

The average raspberry density increased with advancing age. The non-parametric tests demonstrated statistically significant differences in raspberry density when comparing the groups aged 60–99 years and 70–99 years to those aged 20–29 years (*P* < 0.012) and 30–59 years (*P* < 0.011), respectively. The multiple linear regression models demonstrated positive associations with age interval (*P* < 0.001), cerebral atherosclerosis (*P* = 0.024), cardiac hypertrophy (*P* = 0.021), hypertension subgrouped for organ damage (*P* = 0.006), and female sex (*P* = 0.004), and a tendency towards a negative association with Alzheimer’s disease neuropathologic change (*P* = 0.048).

**Conclusion:**

The raspberry density of the frontal cortex increases with advancing age, but our results also indicate associations with acquired pathologies. Awareness of the biological and pathological context where raspberries occur can guide further research on their origin.

## Introduction

**Fig. 1:**
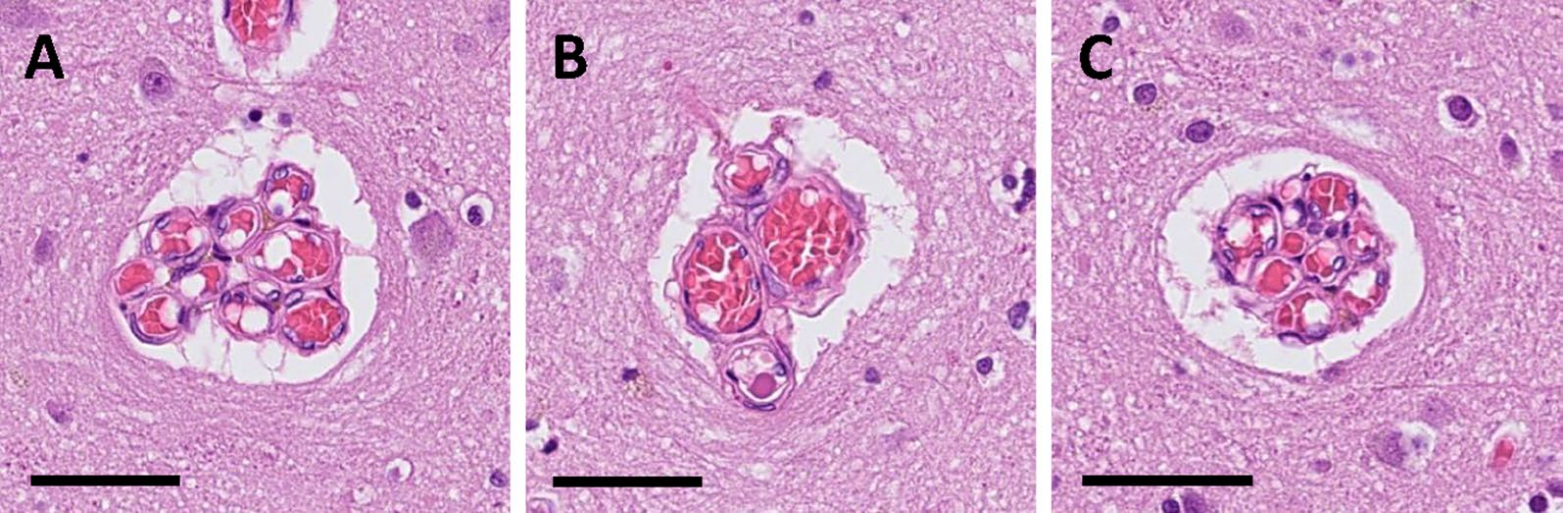
**A–C** Three microvascular raspberries in the frontal cortex. Stain: haematoxylin and eosin. Scalebar: 50 µm

We have previously examined a microvascular formation of the human brain that we termed ‘raspberry’, referring to its appearance under a brightfield microscope (Fig. 1). A raspberry is defined as a minimum of three adjacent microvascular lumina surrounded by a common perivascular space and sectioned transversally [1–3]. According to our observations, raspberries occur mainly in cortical and subcortical grey matter and are of arteriolar calibre [3], although a co-existing capillary or venular component has not been systematically excluded. We have previously hypothesised that raspberries could be a sign of chronic or recurrent hypoperfusion, but their origin remains obscure.

Since the 1960s, cortical microvascular formations that share at least a partial overlap with raspberries have been visualised with brightfield microscopy, microangiography, and electron microscopy; they have often been referred to as vascular ‘wickerworks’, ‘bundles’ or ‘convolutes’ [4–13] (for a brief review on observations prior to the 1960’s, see [4]). While some of these observations were secondary findings in studies of the microvascular architecture, few attempts have been made to examine the formations systematically. These attempts include the works of Hassler [4, 5], Cervos-Navarro et al. [11], Arsene et al. [12, 13], and recently Ighodaro et al., who quantified vascular formations—defined in the same manner as raspberries but referred to as ‘multi-lumen vascular profiles’—in immunohistochemically labelled tissue sections from the frontal cortex [14]. A recurring observation among these authors is an association between the studied vascular entity and advancing age [4, 5, 11, 14]; however, with exception of the study by Ighodaro et al. [14], the results did not undergo statistical analysis.

Our previous findings include a higher cortical raspberry density of statistical significance in vascular dementia compared to age-matched individuals with Alzheimer’s disease, frontotemporal lobar degeneration, and cognitively healthy controls [1]. We also demonstrated an association between cortical raspberry density and atherosclerosis of the basal cerebral arteries, but this finding was weaker than originally indicated when tested in an independent study sample that had been matched for age and sex [2, 3]. Our previous observations on the relation between raspberry density and age have been statistically inconclusive, but our studies had other primary aims and did not include individuals under age 46 [1–3]. Determining the extent of an independent association between raspberries and age would improve our understanding of these vascular formations. As such, the aim of the present work was to examine the raspberry density of the frontal cortex in a large and well-characterised study sample with a wide age range, while including several potential confounding factors in the analysis.

## Materials and methods

### Study population

The present study was a retrospective cross-sectional study based on the examination of tissue sections, autopsy reports, and medical records of adult patients who had undergone a clinical autopsy including a diagnostic neuropathological examination at the Department of Clinical Pathology (Sektion Klinisk Patologi), Region Skane, Lund. The study population consisted of individuals from two previous studies on raspberries and newly included individuals (summarised in Table 1). One of the previous studies was based on consecutively received cases and sought no specific diagnosis [2], while the other study included patients based on their status regarding cerebral atherosclerosis and acute circulatory failure, as documented in the autopsy reports and medical records, respectively [3]. A total of 193 patients were included from these two studies.

**Table 1:** The study population consisted of individuals from two previous publications and newly included individuals, as summarised here

Publication	n	Age range	Inclusion period	Study population
Ek Olofsson et al. [2]	62	46–97	April 2019–January 2021	Patients who had undergone a neuropathological examination where tissue sections from ≥ 10 different brain regions had been sampled—including a tissue section from the anterior frontal lobe—and the brain weight had been documented. Furthermore, a complete diagnostic autopsy report and access to clinical data from the medical records was required. No specific diagnosis was sought
Ek Olofsson et al. [3]	131*	57–92	January 1993–September 2021	Patients that could be fit into one of three groups based on their status regarding cerebral atherosclerosis and acute circulatory failure and matched with patients from the other two groups according to age, sex, and examined brain region
Newly included	70	20–59	January 2013–April 2023	Patients who were 20–59 years old at the time of death and had undergone a neuropathological examination in which one of the sampled tissue sections were from the frontal lobe
Total	263	20–97	January 1993–April 2023	Adult patients who had undergone a clinical autopsy including a diagnostic neuropathological examination at the Section of Clinical Pathology, Region Skane, Lund

*The original study sample consisted of 141 cases. Of these, fifteen had had their cortical raspberry density quantified in another region than the frontal lobe. In five of these cases, a tissue section from the frontal lobe was available, and the raspberry density could be measured retrospectively in this region. The remaining ten cases were excluded

The newly included individuals were autopsied between January 2013 and April 2023, were 20–59 years old at the time of death and had undergone a diagnostic neuropathological examination in which one of the sampled tissue sections was from the frontal lobe. The inclusion was carried out in reverse chronological order and continued until all cases from the period had been assessed, or until a maximum of 25 patients within a given 10-year interval had been included (for example, 50–59 years of age). This approach resulted in the inclusion of 70 patients.

**Fig. 2:**
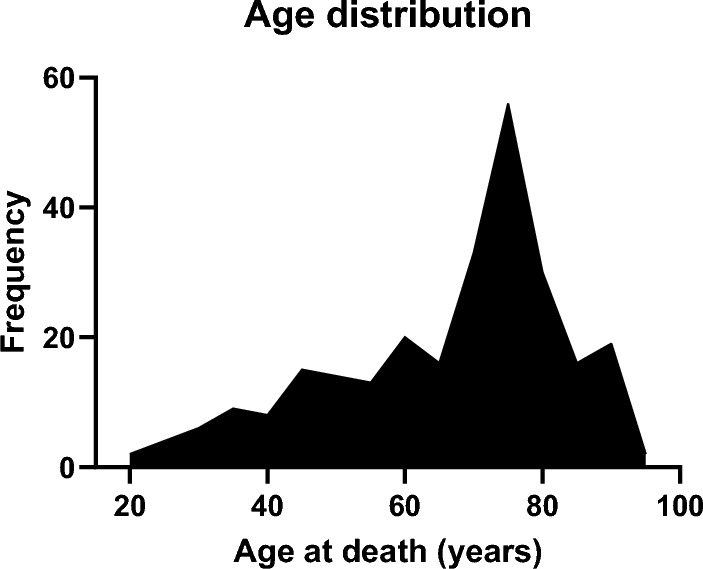
The frequency distribution of age within the study sample (n = 263)

In total, the study population consisted of 263 individuals between ages 20 and 97 (the age distribution is illustrated in Fig. 2).

### Histopathological procedure

All biological material was retrieved as part of the clinical diagnostic process, which was carried out prior to the start of the study. To summarise the histopathological procedure, the whole brain was fixed in formalin for several weeks, after which the forebrain was cut into 1.5-cm thick coronal sections, and the cerebellum and brainstem were cut into 0.5-cm thick horizontal sections. After macroscopic examination, selected tissue blocks were sampled for dehydration and paraffin embedding, followed by microtome cutting and staining. The standard sampling of tissue blocks for the diagnostics of dementias has been described elsewhere [15]; the sampling in other cases was similar but could be modified depending on the specific clinical context.

### Raspberry quantification

**Fig. 3:**
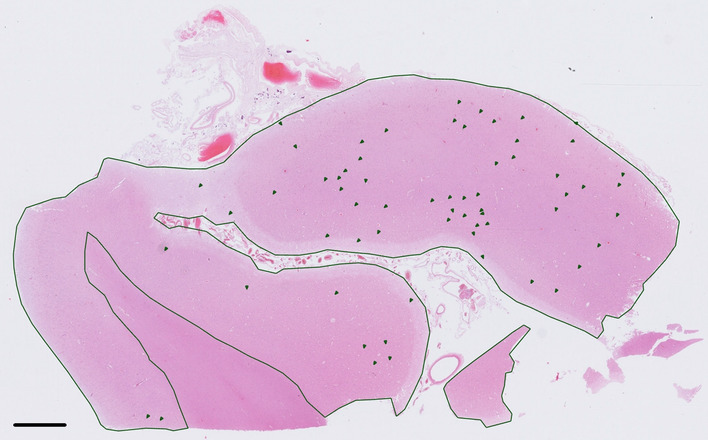
Overview of a tissue section from the frontal lobe. The cortical area has been delineated and the observed raspberries have been marked. Stain: haematoxylin and eosin. Scalebar: 2 mm

The quantification of raspberries was performed manually on 6-µm thick tissue sections from the frontal lobe (Brodmann area 10, 9, or 46) that had been stained with haematoxylin and eosin and mounted on standard-sized glass slides (an example is shown in Fig. 3). The decision to examine the frontal cortex was based on our earlier findings of an accentuated raspberry density in this region [1]; it is also comparable with the sampling applied by other authors [14]. Tissue sections collected prior to April 2019 were viewed and examined in Aperio ImageScope, while later collected tissue sections were examined in Sectra IDS7. The raspberry quantification had either taken place during a previous study or was carried out de novo, blinded to data on patient characteristics. All measurements were performed uniformly as described previously [2, 3]; in summary, cortical structures identified as raspberries were noted, the cortical area was measured, and the raspberry density was documented as raspberries/cm^2^. As a minor modification compared to the previous studies, the raspberry density was not standardised for variations in brain weight.

### Data from medical records and autopsy reports

Autopsy reports prior to April 2019 were accessed through Sympathy, while later reports were accessed through LIMS RS; these are digital databases with records of all autopsies performed in Region Skane. The medical records were accessed via the digital system Melior, where public specialist healthcare in Region Skane is documented. The status of each finding was categorised as ‘present’, ‘absent’, or ‘missing/indeterminate’ unless otherwise indicated. The variables are listed in the following sections (further details regarding their definitions are provided in Table 2).

**Table 2:** Variables retrieved from autopsy reports and medical records, and their definitions

Variable	Retrieved from	Definition
Alzheimer's disease neuropathologic change	Autopsy report	Alzheimer's disease neuropathologic change with neurofibrillary tangle pathology corresponding to Braak stage III or more [16–18]
Lewy body disease or multiple system atrophy	Autopsy report	Lewy body disease of brainstem-predominant, limbic, or neocortical category [18–20]. Multiple system atrophy regardless of subtype [21, 22]
Frontotemporal lobar degeneration	Autopsy report	Frontotemporal lobar degeneration regardless of subtype [23–25]
Vascular cognitive impairment	Autopsy report and medical records	Multiple ischaemic lesions, absence of relevant neurodegenerative disease, and symptoms suggestive of mild cognitive impairment or dementia [15, 26]
Mixed/other neurodegenerative disease	Autopsy report	Neurodegenerative disease co-occurring with multiple infarcts, combined neurodegenerative diseases, or other/unclassifiable neurodegenerative pathology
Cerebral infarcts	Autopsy report	Ranked ordinally as none, one, or multiple
Cerebral atherosclerosis	Autopsy report	Atherosclerotic plaques in the basilar artery or the circle of Willis
Cerebral small vessel disease	Autopsy report	Cerebral arteriolosclerosis of hyaline or hyperplastic type [27]
Cardiac hypertrophy	Autopsy report	Heart weights suggestive of hypertrophy were calculated in retrospect according to the model by Vanhaebost et al. [28]. Based on average heights and body weights of the Swedish population [29], a heart weight exceeding 386 g in women (166 cm, 68 kg) or 463 g in men (180 cm, 84 kg) was considered suggestive of hypertrophy
Nephrosclerosis	Autopsy report	Macroscopic granular scarring and/or microscopic arteriolosclerosis and glomerulosclerosis [30, 31]
Hypertension	Medical records	Clinically diagnosed
Hypertensive organ damage	Autopsy report and medical records	In individuals diagnosed with hypertension, cardiac hypertrophy in combination with either nephrosclerosis or cerebral small vessel disease was considered suggestive of hypertensive organ damage [32]. In absence of clinically diagnosed hypertension, cases with this combination of organ findings were categorised as indeterminate
Diabetes mellitus	Medical records	Clinically diagnosed

From the autopsy reports, the status of the following variables was retrieved: Alzheimer’s disease neuropathologic change, Lewy body disease or multiple system atrophy, frontotemporal lobar degeneration, vascular cognitive impairment, mixed/other neurodegenerative disease, cerebral infarcts (ranked ordinally as ‘none’, ‘one’ or ‘multiple’), cerebral small vessel disease, cerebral atherosclerosis, brain weight (g), cardiac hypertrophy, and nephrosclerosis. Other significant neuropathological findings were noted but not included in the statistical analyses (demyelinating disease, malformation, malignancy, toxic disease, traumatic injury).

From the medical records, information was retrieved regarding hypertension and diabetes mellitus. In cases of vascular mild cognitive impairment and vascular dementia, the cognitive status could be assessed and verified via the medical records. Concerning the patients with neurodegenerative disease, most had shown clinical symptoms suggestive of such pathology, but the definitions of these variables were based on the findings of the neuropathological examination, regardless of the availability of clinical information.

### Statistics

IBM SPSS Statistics Version 28 was used to perform the statistical analyses. The patients were grouped based on 10-year intervals, from 20–29 years of age to 90–99 years of age, resulting in an ordinal variable termed ‘age interval’. Differences in raspberry density according to age interval were analysed using non-parametric testing (the Kruskal–Wallis test, followed by Dunn’s post hoc test with Bonferroni-corrected *P* values) and parametric testing (multiple linear regression). The first multiple linear regression model included the following independent variables (defined as nominal unless otherwise indicated): age interval (ordinal), sex, brain weight (continuous), Alzheimer’s disease neuropathologic change, Lewy body disease or multiple system atrophy, frontotemporal lobar degeneration, vascular cognitive impairment, mixed/other neurodegenerative disease, cerebral infarcts (ordinal), cerebral atherosclerosis, cerebral small vessel disease, cardiac hypertrophy, nephrosclerosis, hypertension, and diabetes mellitus. To exclude a potential additive effect, a second analysis was performed while defining all neurodegenerative disease and vascular cognitive impairment as a single entity and ranking hypertension ordinally based on the presence or absence of hypertensive organ damage. A *P* value of ≤ 0.05 was considered statistically significant.

## Results

The study population consisted of 263 individuals with an age range of 20–97 years and a median age of 71 years. 32% of the patients were female. The sample exhibited a high prevalence of neurodegenerative disease with an uneven distribution across the age range (patient characteristics including demographics, neuropathological findings, other organ findings, and clinical diagnoses are presented in Table 3).

**Table 3:** Patient characteristics in relation to age interval

Age interval	20–29	30–39	40–49	50–59	60–69	70–79	80–89	90–99	Total
n	9	14	23	37	32	100	37	11	263
Female sex	4/9 (44%)	6/14 (43%)	10/23 (43%)	12/37 (32%)	12/32 (38%)	27/100 (27%)	11/37 (30%)	1/11 (9%)	83/263 (32%)
Median brain weight	1270 g	1430 g	1400 g	1410 g	1343 g	1303 g	1269 g	1273 g	1320 g
ADNC	0/9 (0%)	0/14 (0%)	1/23 (4%)	0/37 (0%)	3/32 (9%)	7/100 (7%)	6/37 (16%)	4/11 (36%)	21/263 (8%)
LBD or MSA	0/9 (0%)	0/14 (0%)	0/23 (0%)	4/37 (11%)	3/32 (9%)	12/100 (12%)	1/37 (3%)	0/11 (0%)	21/263 (8%)
FTLD	0/9 (0%)	0/14 (0%)	0/23 (0%)	4/37 (11%)	4/32 (13%)	17/100 (17%)	4/37 (11%)	0/11 (0%)	29/263 (11%)
VCI	0/9 (0%)	0/14 (0%)	0/23 (0%)	1/37 (3%)	1/32 (3%)	10/100 (10%)	5/37 (14%)	3/11 (27%)	21/263 (8%)
Mixed/other NDD	0/9 (0%)	0/14 (0%)	0/23 (0%)	0/37 (0%)	5/32 (16%)	20/100 (20%)	13/37 (35%)	3/11 (27%)	41/263 (16%)
NDD and VCI, total	0/9 (0%)	0/14 (0%)	1/23 (4%)	9/37 (24%)	16/32 (50%)	66/100 (66%)	29/37 (78%)	10/11 (91%)	131/263 (50%)
CVI, one	0/9 (0%)	1/14 (7%)	3/23 (13%)	3/36 (8%)	2/31 (6%)	15/97 (15%)	6/35 (17%)	4/11 (36%)	34/256 (13%)
CVI, multiple	2/9 (22%)	0/14 (0%)	1/23 (4%)	5/36 (14%)	14/31 (45%)	34/97 (35%)	14/35 (40%)	5/11 (45%)	75/256 (29%)
CVI, total	2/9 (22%)	1/14 (7%)	4/23 (17%)	8/36 (22%)	16/31 (52%)	49/97 (51%)	20/35 (57%)	9/11 (82%)	109/256 (43%)
C-ASCL	0/8 (0%)	0/12 (0%)	3/21 (14%)	6/35 (17%)	12/32 (38%)	57/97 (59%)	20/37 (54%)	7/11 (64%)	105/253 (42%)
C-SVD	0/9 (0%)	0/14 (0%)	6/22 (27%)	10/37 (27%)	10/32 (31%)	47/97 (48%)	22/36 (61%)	4/10 (40%)	99/257 (39%)
Cardiac hypertrophy	0/5 (0%)	0/7 (0%)	5/14 (36%)	6/19 (32%)	14/30 (47%)	39/94 (41%)	16/36 (44%)	6/10 (60%)	86/215 (40%)
Nephrosclerosis	0/7 (0%)	0/10 (0%)	2/18 (11%)	2/31 (6%)	8/31 (26%)	51/100 (51%)	23/37 (62%)	8/11 (73%)	94/245 (38%)
HT, no organ damage	0/9 (0%)	0/13 (0%)	2/21 (10%)	4/37 (11%)	11/31 (35%)	31/89 (35%)	12/37 (32%)	3/9 (33%)	63/246 (26%)
HT, organ damage	0/9 (0%)	0/13 (0%)	2/21 (10%)	2/37 (5%)	6/31 (19%)	19/89 (21%)	10/37 (27%)	2/9 (22%)	41/246 (17%)
HT, unclassified	0/9 (0%)	0/13 (0%)	1/21 (5%)	6/37 (16%)	0/31 (0%)	5/89 (6%)	1/37 (3%)	0/9 (0%)	13/246 (5%)
HT, total	0/9 (0%)	0/13 (0%)	5/21 (24%)	12/37 (32%)	17/31 (55%)	55/89 (62%)	23/37 (62%)	5/9 (56%)	117/246 (48%)
Diabetes mellitus	0/9 (0%)	0/13 (0%)	5/23 (22%)	6/37 (16%)	9/29 (31%)	21/90 (23%)	10/36 (28%)	3/11 (27%)	54/248 (22%)
Other findings*	4/9 (44%)	3/14 (21%)	6/23 (26%)	7/37 (19%)	1/32 (3%)	7/100 (7%)	1/37 (3%)	0/11 (0%)	29/263 (11%)

*Neuropathological findings not included in the statistical analyses: demyelinating disease (n = 6), malformation (n = 3), malignancy (n = 5), toxic disease (n = 5), traumatic injury (n = 3), other/unclassified (n = 7). ADNC Alzheimer's disease neuropathologic change; LBD Lewy body disease; MSA multiple system atrophy; VCI vascular cognitive impairment; NDD neurodegenerative disease; CVI cerebral infarct; C-ASCL cerebral atherosclerosis; C-SVD cerebral small vessel disease; HT hypertension

**Fig. 4:**
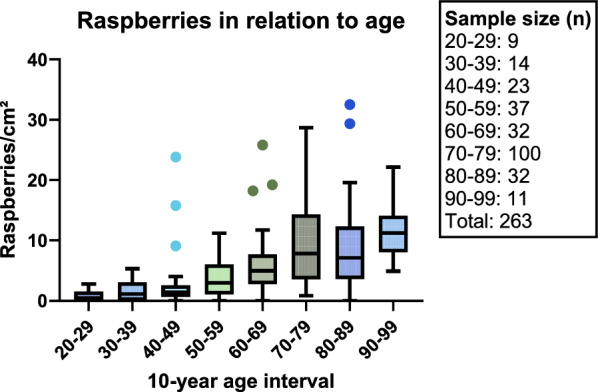
Raspberry density (raspberries/cm^2^) of the frontal cortex in relation to 10-year age interval. With the sample grouped according to the graph, pairwise testing (Dunn’s post hoc test with Bonferroni-corrected *P* values; Table 5) indicated differences between the groups aged 20–59 years and those aged 70–99 years; the group aged 20–29 years also differed from the one aged 60–69 years

The mean and median raspberry density of the frontal cortex increased with advancing age, with the exception of a slight decrease between the 70–79 age group and the 80–89 age group (Table 4 and Fig. 4). The Kruskal–Wallis test exhibited a statistically significant difference in raspberry density according to age interval (*P* < 0.001), and consequently, pairwise tests were run (Table 5). After Bonferroni correction, these tests demonstrated statistically significant differences in raspberry density when comparing any age group from the 20–59 interval to any age group from the 70–99 interval. The raspberry density in the 20–29 age group also differed significantly, after Bonferroni correction, when compared to the 60–69 age group.

**Table 4:** Raspberry density of the frontal cortex in relation to age interval

Age interval	n	Raspberry density (raspberries/cm^2^)		
		Mean	Median	Range
20–29	9	0.8	0.5	0.0–2.8
30–39	14	1.8	1.1	0.0–5.3
40–49	23	3.3	1.4	0.0–23.8
50–59	37	3.8	3.0	0.0–11.2
60–69	32	6.5	5.0	0.0–25,8
70–79	100	9.4	7.8	0.9–28.7
80–89	37	9.2	7.1	0.0–32.5
90–99	11	11.3	11.2	4.9–22.1
Total	263	7.0	5.4	0.0–32.5

**Table 5:** Results of the pairwise testing (Dunn’s post hoc test) that followed the statistically significant (*P* < 0.001) Kruskal–Wallis test

Age interval	20–29 (n = 9)	30–39 (n = 14)	40–49 (n = 23)	50–59 (n = 37)	60–69 (n = 32)	70–79 (n = 100)	80–89 (n = 37)
30–39 (n = 14)	1.00 (0.45)	–					
40–49 (n = 23)	1.00 (0.19)	1.00 (0.58)	–				
50–59 (n = 37)	0.66 (0.024)	1.00 (0.10)	1.00 (0.22)	–			
60–69 (n = 32)	**0.012** (< 0.001)	0.051 (0.002)	0.085 (0.003)	1.00 (0.045)	–		
70–79 (n = 100)	**< 0.001** (< 0.001)	**< 0.001** (< 0.001)	**< 0.001** (< 0.001)	**< 0.001** (< 0.001)	0.95 (0.034)	–	
80–89 (n = 37)	**< 0.001** (< 0.001)	**0.001** (< 0.001)	**< 0.001** (< 0.001)	**0.011** (< 0.001)	1.00 (0.16)	1.00 (0.64)	–
90–99 (n = 11)	**< 0.001** (< 0.001)	**< 0.001** (< 0.001)	**< 0.001** (< 0.001)	**0.003** (< 0.001)	0.39 (0.014)	1.00 (0.13)	1.00 (0.18)

The table shows Bonferroni-corrected *P* values, followed by unadjusted *P* values within parentheses. Statistically significant *P* values are marked in bold. Outcome variable: raspberry density (raspberries/cm^2^) of the frontal cortex

**Fig. 5:**
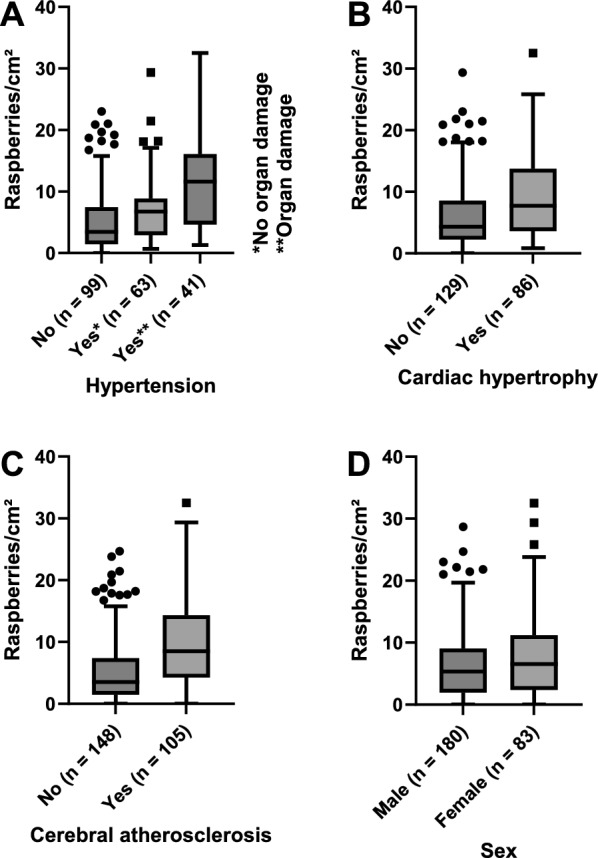
Raspberry density (raspberries/cm^2^) of the frontal cortex in relation to **A** hypertension subgrouped for organ damage, **B** cardiac hypertrophy, **C** cerebral atherosclerosis, and **D** sex. Multiple linear regression models indicated independent associations between these variables and raspberry density (Table 6 and Table 7; for definitions of the variables, see Table 2)

The results of the first multiple linear regression model are presented in Table 6. There was a positive association between cortical raspberry density and age interval at a statistically significant level (*P* < 0.001). Other statistically significant results included a positive association with female sex (*P* = 0.004), cerebral atherosclerosis (*P* = 0.015), and cardiac hypertrophy (*P* = 0.021), and a tendency for a negative association with Alzheimer’s disease neuropathologic change (*P* = 0.048). In the second multiple linear regression model, all neurodegenerative disease and vascular cognitive impairment were defined as a single entity, and hypertension was subgrouped based on the presence or absence of hypertensive organ damage. The results of this analysis indicated a positive association between subgrouped hypertension and raspberry density (*P* = 0.006); the associations with age interval (*P* < 0.001), female sex (*P* = 0.004), and cerebral atherosclerosis (*P* = 0.024) all remained statistically significant (Table 7). Descriptive data regarding the raspberry density in relation to selected variables is presented in Fig. 5A–D.

**Table 6:** *P* values from the first multiple linear regression model

Independent variable	*P* value	95% CI
Age interval*	**< 0.001**	**+ 0.6, + 2.2**
Female sex	**0.004**	**+ 1.0, + 5.3**
Brain weight**	0.56	NS
ADNC	**0.048**	**− 7.0, − 0.03**
LBD or MSA	0.86	NS
FTLD	0.99	NS
VCI	0.073	NS
Mixed/other NDD	0.32	NS
Cerebral infarct*	0.17	NS
C-ASCL	**0.015**	**+ 0.5, + 4.7**
C-SVD	0.40	NS
Cardiac hypertrophy	**0.021**	**+ 0.3, + 4.3**
Nephrosclerosis	0.63	NS
Hypertension	0.97	NS
Diabetes mellitus	0.89	NS

Outcome variable: raspberry density (raspberries/cm^2^) of the frontal cortex. Statistically significant results are marked in bold and accompanied by 95% confidence intervals for the beta coefficients. The independent variables were classified as nominal unless otherwise indicated. *Ordinal. **Continuous. CI confidence interval; NS not significant; ADNC Alzheimer’s disease neuropathologic change; LBD Lewy body disease; MSA multiple system atrophy; FTLD frontotemporal lobar degeneration; VCI vascular cognitive impairment; NDD neurodegenerative disease; C-ASCL cerebral atherosclerosis; C-SVD cerebral small vessel disease

**Table 7:** *P* values from the second multiple linear regression model, in which all neurodegenerative disease and vascular cognitive impairment were defined as a single entity, and hypertension was subgrouped based on the presence or absence of hypertensive organ damage

Independent variable	*P* value	95% CI
Age interval*	**< 0.001**	**+ 0.5, + 1.9**
Female sex	**0.004**	**+ 0.9, + 4.8**
Brain weight**	0.77	NS
NDD and VCI, total	0.91	NS
Cerebral infarct*	0.36	NS
C-ASCL	**0.024**	**+ 0.3, + 4.3**
HT, subgrouped*	**0.006**	**+ 0.5, + 3.2**
Diabetes mellitus	0.35	NS

Outcome variable: raspberry density (raspberries/cm^2^) of the frontal cortex. Statistically significant results are marked in bold and accompanied by 95% confidence intervals for the beta coefficients. The independent variables were classified as nominal unless otherwise indicated. *Ordinal. **Continuous. CI confidence interval; NS not significant; VCI vascular cognitive impairment; C-ASCL cerebral atherosclerosis; HT hypertension

## Discussion

### General comments on study design

The aim of the present study was to examine the relation between raspberries and advancing age, including to what extent this association can be considered independent. Our study sample consisted of individuals from two previous studies as well as newly included individuals. Apart from cerebral atherosclerosis and acute circulatory failure in one of the previous studies [3], no specific diagnoses had been sought, and all patients were included regardless of other pathologies, resulting in a high prevalence of neurodegenerative disease in particular. A study sample representative of the general population would have been difficult to obtain regardless of the selection criteria, but a more representative sample could likely have been achieved by a stricter inclusion. However, such a sample also would have been smaller and at higher risk of being underpowered, thus reducing the chances of identifying small but pertinent associations with other variables than with the most influential ones. Further, the retrospective nature of the study is a limitation because it makes access to data and categorisation of variables less reliable and reduces the opportunity to semi-quantify the data. Such limitations can be partially overcome by expanding the study sample and can be further handled—or otherwise visualised—by using the full range of data to characterise the sample, including clinical data, neuropathological findings, and findings in other organs.

The differences in inclusion criteria between the previously and newly included individuals may have affected the proportion of patients with cerebral atherosclerosis within the study sample. As such, this finding was included as one of the potential confounding factors in the multiple linear regression models. In contrast, the influence of acute circulatory failure was assumed to be negligible, based on our earlier results [3]. Unlike our previous studies, we did not adjust the raspberry density for deviations from normal brain weight. Such standardisation was originally performed under the assumption that altered brain volume caused by atrophy or oedema—and manifested as an abnormal brain weight—could affect the raspberry density in a way that did not reflect a change in the absolute number of raspberries. However, altered volume may not be the only possible explanation for an increased raspberry density in an atrophic brain. As such, adjusting for brain weight may be a valid approach if weight variations within the sample can be assumed to be random in relation to the grouping variable. In contrast, such standardisation could have risked obscuring the results of the current study due to age-related atrophy or acquired pathologies that become more prevalent among the elderly. Consequently, we separated brain weight from our outcome variable (raspberry density) and instead included brain weight as a variable in the multiple linear regression models.

### Raspberries and age

The main finding of the present study is that the raspberry density of the frontal cortex increases with advancing age, as demonstrated by parametric and non-parametric testing. The results are in line with studies on overlapping and synonymous vascular entities [4, 5, 11, 14]. Our previous studies were unable to demonstrate a statistically significant age-dependent variation in raspberry density, but as stated in the introduction, these studies did not include subjects younger than 46 years, nor did they compare as many subjects as in the present study. Due to a skewed outcome variable with an unequal variance between the groups, the non-parametric model is more accurate at explaining the relation between raspberry density and age—that older individuals are more likely than younger individuals to have a high raspberry density. However, we ran multiple linear regression in tandem to enable an examination of confounding factors.

The non-parametric tests indicate that the major increase in average raspberry density occurs in the 60–69 or 70–79 interval. Due to the burden of various pathologies within the study sample—especially neurodegenerative disease within the older age groups—some caution is required when attempting to generalise these results. While the multiple linear regression models did not indicate neurodegenerative disease or vascular cognitive impairment as confounding factors, the results do not exclude that these diagnoses have an independent impact on raspberry density (doing this would require a study designed with a different primary aim in mind [1]). Consequently, while we conclude that there is an association between cortical raspberry density and advancing age, the external validity regarding the specific distribution of raspberry density across the age range may be limited by the high prevalence of neurodegenerative disease in the study sample, and possibly by the occurrence of other pathologies. With this caveat in mind, the distribution appears to be similar to that reported by Ighodaro et al. regarding the synonymous multi-lumen vascular profiles—in a sample in which cases with end-stage neurodegenerative diseases had been excluded [14]—and by Hassler regarding the overlapping vascular ‘bundles’ and ‘wickerworks’—in a series of consecutively received cases [5].

The finding that cortical raspberries occurred in all age groups contrasts observations on overlapping vascular entities, which were not reported in young adults [4, 5, 11]. The presence of cortical raspberries among the youngest individuals in our sample may have been the result of unknown pathological factors, since neuropathological findings not included in the statistical analyses occurred in our sample, albeit infrequently regarding the individual diagnoses. Another possibility is that a small number of cortical raspberries is a normal physiological finding, and that potential reference values for the ‘baseline’ raspberry density in the adult population should not be limited to zero. Potentially in favour of this possibility is that the recent study by Ighodaro et al. [14] reported the occurrence of ‘multi-lumen vascular profiles’ at a low density in the frontal cortex of young adults. The definition of multi-lumen vascular profiles can be considered synonymous with the definition of raspberries, and the methods of quantification are highly similar. As such, it is not surprising that our results agree in this regard, while the findings of other authors could be affected by differences in definitions and methodologies. Unlike Ighodaro et al. [14], we did not use immunohistochemical labelling of blood vessels to visualise the raspberries. However, while the application of immunohistochemistry can reveal a larger number of raspberries than haematoxylin and eosin, the latter can still enable the distinction of relative differences [1].

The association between raspberry density and age interval raises the question of whether raspberry formation beyond ‘baseline’ is caused by age-related effects on the vasculature. On one hand, ageing may be a marker for a combination of acquired pathologies that together contribute to raspberry formation, but where the influence of each individual pathology is minor and difficult to distinguish. Alternatively, normal ageing itself could impact on raspberry formation. According to one model of ageing, oxidative stress and other forms of cumulative cellular damage induce senescence and additional responses to injury that ultimately become deleterious by contributing to stem cell exhaustion, impaired cell-to-cell interaction, and chronic inflammation [33, 34]. As such, ageing increases the risk for a wide range of disorders, including atherosclerosis, hypertension, and neurodegeneration, while also exerting an independent influence on the structure and function of tissues and organs [33, 34]. From a vascular perspective, an important manifestation of ageing is the remodelling and stiffening of elastic arteries that predisposes for hypertension and increased pulse pressure; these proximal alterations are thought to predate and progress the remodelling of the micro-vessels [35, 36]. Microvascular remodelling includes arteriolosclerosis, increased arteriolar length and tortuosity, thickening of the capillary basement membrane, and rarefaction of arterioles and capillaries [37, 38]. Altered neurovascular coupling, compromised integrity of the blood–brain barrier, and reduced angiogenic capacity constitute functional consequences [36, 39]. Chronic inflammation may also affect the angiogenic threshold, depending on the balance between pro- and antiangiogenic inflammatory cytokines [40]. Effects on cerebral blood flow include decreasing total blood flow to the cerebral cortex [41], a decline that may be the most pronounced after age 60 [42]. Consensus has not been reached on whether all these changes are deleterious or at least partially caused by reduced metabolic needs [36]. However, the alterations briefly covered here may provide some indication of the biological context where raspberries and similar structures can be hypothesised to occur, given the increasing raspberry density with advancing age, and the results by Ighodaro et al. on synonymous formations [14]. Additional research is required to accurately place raspberries within this context. Such future studies may encompass the testing of additional clinicopathological correlations, the application of other techniques such as immunohistochemistry, transcriptomics, and proteomics, or experimental approaches.

### Raspberries and acquired pathologies

However, effects other than the direct impact of ageing must be accounted for. In addition to age, the multiple linear regression models demonstrated positive associations between raspberry density and cerebral atherosclerosis, cardiac hypertrophy, and hypertension subgrouped for organ damage. There was also a positive association with female sex and a tendency towards a negative association with Alzheimer’s disease neuropathologic change. Some of these findings are discussed in the following sections, while keeping in mind that the lack of correction for multiple tests gives this part of the study an exploratory nature.

There was a positive association between raspberry density and hypertension subgrouped for organ damage. These results indicate that the raspberry density can be positively associated with hypertension if the severity of the hypertension is accounted for. In the current study, the severity was ranked by retrieving data on findings suggestive of hypertensive organ damage from the autopsy reports, including cardiac hypertrophy, nephrosclerosis, and cerebral small vessel disease. While not tested in our study, one could speculate on an interaction between hypertension and ageing—and possibly other risk factors—for raspberry formation. Ageing is associated with arterial stiffening that is thought to predate and contribute to remodelling of the microvasculature; hypertension, in turn, is thought to accelerate this process [35, 36]. Perhaps raspberries are a sign of microvascular remodelling that occurs prior to histopathologically manifest small vessel disease—a variable that was not independently associated with raspberry density in our material. The fact that the retrospective study design did not admit optimal definitions of the variables is a limitation also in the assessment of small vessel disease, but the lack of an association does not contradict the findings of other authors [14]. However, the lack of an association between raspberry density and hypertension when not subgrouping for organ damage illustrates that the results should not be overinterpreted, and further studies that focus on the clinical aspect of this association are desirable.

We also found an association between raspberry density and cardiac hypertrophy. This variable was defined in retrospect based on heart weight and was one of the variables used to define hypertensive organ damage. We applied the model by Vanhaebost et al. [28] to define heart weights suggestive of hypertrophy, as their data is relatively new, based on a Caucasian population, and aimed at establishing reference values for normal, healthy heart weights. Since heart weight can be increased for reasons other than hypertension, our finding of an association between raspberry density and isolated cardiac hypertrophy is relatively non-specific but potentially indicative of cardiac pathology. While exploratory, our results on subgrouped hypertension and cardiac hypertrophy indicate that extracranial pathology can be an important component in the mapping of raspberries.

The positive association between raspberry density and cerebral atherosclerosis is in line with our earlier findings. However, the study sample was not independent, since the samples wherein the association was originally examined were both included in the present work [2, 3]. Of note, our previous data indicate that a relatively large study sample is required to examine this association with satisfying statistical power [3].

Relating our findings to those of other authors, weak associations between vascular bundles/wickerworks and cardiac hypertrophy as well as cerebral atherosclerosis were described by Hassler, but he did not report a statistical analysis of his data [5]. Additionally, the findings of Arsene et al. led them to propose a hypertensive aetiology for the similarly defined ‘vascular convolutes’, but the statistical analyses that tested this hypothesis directly were inconclusive [13].

There was a slightly higher raspberry density in women at a statistically significant level. To our knowledge, such an association has not been reported by other authors but is in line with the descriptive data in one of the previous studies from which the current study population was drawn [2]. Considering the age distribution, most of the women included in the study can be assumed to have undergone menopause; whether this fact is of any significance cannot be further analysed with our current data. Despite the statistically significant results after multiple linear regression, it may not be possible to exclude selection bias caused by differing reasons for admitting men and women to autopsy. However, the results may indicate that variations in sex distribution should be accounted for in future studies, in addition to age.

## Conclusion

This study demonstrates an increasing raspberry density in the frontal cortex with advancing age that remains statistically significant after adjusting for multiple potential confounding factors. Moreover, our results indicate positive associations with cerebral atherosclerosis, cardiac hypertrophy, hypertension subgrouped for organ damage, and female sex; due to the retrospective and exploratory nature of the current work, studies that address these associations specifically would be valuable when further mapping the epidemiology of this vascular entity. Based on our results, the impact of the ageing process as well as acquired vascular pathologies should be considered possible contributors to raspberry formation.

## Data Availability

The datasets used and analysed during the current study are available from the corresponding author on reasonable request.

## Abbreviations

ADNC: Alzheimer’s disease neuropathologic change
C-ASCL: Cerebral atherosclerosis
CI: Confidence interval
C-SVD: Cerebral small vessel disease
CVI: Cerebral infarct
FTLD: Frontotemporal lobar degeneration
HT: Hypertension
LBD: Lewy body disease
MSA: Multiple system atrophy
NDD: Neurodegenerative disease
NS: Not significant
VCI: Vascular cognitive impairment
